# Constitutive Models for the Tensile Behaviour of TRM Materials: Literature Review and Experimental Verification

**DOI:** 10.3390/ma14030700

**Published:** 2021-02-02

**Authors:** Maria Concetta Oddo, Giovanni Minafò, Lidia La Mendola

**Affiliations:** Dipartimento di Ingegneria, Università degli Studi di Palermo, Viale delle Scienze, 90128 Palermo, Italy; mariaconcetta.oddo01@unipa.it (M.C.O.); giovanni.minafo@unipa.it (G.M.)

**Keywords:** Textile Reinforced Mortar (TRM), tensile behaviour, strengthening

## Abstract

In recent years, the scientific community has focused its interest on innovative inorganic matrix composite materials, namely TRM (Textile Reinforced Mortar). This class of materials satisfies the need of retrofitting existing masonry buildings, by keeping the compatibility with the substrate. Different recent studies were addressed to improve the knowledge on their mechanical behaviour and some theoretical models were proposed for predicting the tensile response of TRM strips. However, this task is complex due to the heterogeneity of the constituent materials and the stress transfer mechanism developed between matrix and fabric through the interface in the cracked stage. This paper presents a state-of-the-art review on the existing constitutive models for the tensile behavior of TRM composites. Literature experimental results of tensile tests on TRM coupons are presented and compared with the most relevant analytical models proposed until now. Finally, a new experimental study is presented and its results are used to further verify the reliability of the literature expressions.

## 1. Introduction

In recent times, the technology of inorganic matrix composites, namely FRCM (Fabric Reinforced Cementitious Matrix) or Textile Reinforced Mortar (TRM) materials, has been used in numerous retrofitting and strengthening practical applications and is preferred to polymer-based composites FRPs (Fibre Reinforced Polymers), especially for masonry structures [1]. In fact, the use of inorganic matrices allows TRM systems to include many advantages when applied to an existing masonry support, overcoming the drawbacks associated to the adoption of epoxy resins. In fact, inorganic matrices have a low cost and good compatibility with the masonry substrate, provide better breathability and fire resistance, allow the reversibility of the intervention. Despite the growing spread of TRM materials, the modelling and characterization of the mechanical response of these innovative composites are still open issues, due to the inner mechanical complexity of the stress transfer mechanisms between fabric, matrix and support. For these reasons, several experimental studies were addressed to the mechanical characterization for the homologation and the acceptance of TRM composites, with particular reference to their tensile behaviour. Experimental outcomes have shown that many variables are involved in the tensile constitutive laws, in particular the nature of fabric, eventual treatment of the yarns, the test set-up employed, the amount and the geometry of the fabric and the grade of the mortar.

Despite several experimental studies investigated the tensile behaviour of TRMs, fewer investigations proposed analytical formulations for predicting their constitutive law in tension. Generally, two approaches can be found in the literature: simplified analytical approach or micromodelling numerical methods. These last are often based on mesoscale finite element approaches, such those recently adopted by Nerilli et al. [2], Monaco et al. [3] or Grande and Milani [4], which require strong computational capabilities, resulting more suitable for simulation purposes rather than for design oriented applications. From another hand, simplified models are based on a simplification of the tensile behaviour of the composite. They have the advantage of being simple and easy to be used for practical design, but their validity has never been confirmed extensively, considering that the most of experimental studies were performed recently.

This paper shows a review of existing simplified analytical models for the tensile constitutive behaviour of TRM materials, supported by a new experimental investigation. Three different available analytical models were considered, namely the Aveston–Cooper–Kelly (ACK) Model [5], the model proposed by Minafo and La Mendola [6] and Tension Stiffening Model [7]. An experimental database with the results available in literature, analyzed as a function of tensile test method (clevis-grip method or clamping-grip method) and nature of fabric (carbon, basalt and glass). The existing experimental database was enriched by new experimental results obtained on a glass-TRM, and overall the data are compared with theoretical predictions, giving considerations on the reliability of the formulations and their limits of application.

## 2. Existing Analytical Models for Tensile Behaviour of TRM Materials

As discussed above, three analytical models are here considered for modelling the tensile behaviour of TRM materials: the Aveston–Cooper–Kelly (ACK) Model [5], the model proposed by Minafo and La Mendola [6] and the Tension Stiffening Model [7].

### 2.1. ACK Model

The ACK model (ACK), is based on single and multiple fracture of fibrous composites with brittle matrix. Currently, several literature studies are based on ACK theory for modelling the stress-strain tensile behaviour [8,9] and to predict cracking [10]. The typical tensile response can be simplified with a trilinear curve defined by characteristic points.

In the first stage, matrix is uncracked and perfect bond between matrix and fabric is assumed up to the first cracking stress. This last is defined as it follows:

(1) $$\sigma_{cr,ACK}=\frac{E_{t,I}f_{mt}}{E_{m}}$$

where $f_{mt}$ and $E_{m}$ are respectively the tensile strength and the tensile elastic modulus for the mortar. $E_{t,I}$ is the slope of the first branch defined by the well-known mixture law:

(2) $$E_{t,I}=E_{f}V_{f}+E_{m}V_{m}$$

where $V_{f}$ and $V_{m}$ are the volume fractions respectively for fiber and mortar, and $E_{f}$ is the elastic modulus of the fiber. The corresponding strain $\epsilon_{I,ACK}$ is conveniently calculated by dividing the first cracking stress $\sigma_{cr,ACK}$ for the elastic modulus $E_{t,I}$.

The second stage is characterized by the crack propagation. In this phase, the load is assumed to be constant up to the strain value $\epsilon_{II,ACK}$ calculated as it follows:

(3) $$\epsilon_{II,ACK}=\left(1+0.666\alpha_{e}\right)\frac{f_{mt}}{E_{m}}$$

where $\alpha_{e}$ is an homogenisation coefficient:

(4) $$\alpha_{e}=\frac{E_{m}V_{m}}{E_{f}V_{f}}$$

Finally, in the third stage, when the crack pattern is stabilized the load increases linearly up to the ultimate tensile strain of the fiber $\epsilon_{f,u}$, with a slope equal to:

(5) $$E_{III,ACK}=E_{f}V_{f}$$

### 2.2. Simplified Model Proposed by Minafò and La Mendola

The second model considered is a modification of previous ACK model with similar laws for the three stages, and it is here referred as Simplified Model (sm).

The first stage is defined considering the first crack stress as follows:

(6) $$\sigma_{cr,sm}=\frac{f_{mt}A_{id}}{A_{m}+A_{f}}$$

where $A_{id}$ is the ideal area for a TRM strip homogenized with respect to the ratio *n*:

(7) $$A_{id}=A_{m}+\left(nA_{f}\right)$$

(8) $$n=\frac{E_{f}}{E_{m}}$$

the corresponding strain $\epsilon_{cr,sm}$ is conveniently calculated dividing the tensile strength $f_{mt}$ for the tensile elastic modulus $E_{m}$ of mortar:

(9) $$\epsilon_{cr,sm}=\frac{f_{mt}}{E_{m}}$$

The second stage is defined as in the ACK model with a constant load plateau up to $\epsilon_{II,sm}$, this last evaluated according to Equation (3).

Finally, the ultimate stress is obtained by applying the mixture law:

(10) $$\sigma_{u,sm}=\sigma_{f}V_{f}+\sigma_{m}V_{m}$$

and the corresponding ultimate strain is calculated from the sum:

(11) $$\epsilon_{III,sm}=\epsilon_{II,sm}+\epsilon_{f,u}$$

### 2.3. Tension Stiffening Model

Finally, the Tension Stiffening Model (ts) is an approach, originally proposed for reinforced concrete members in tension [7].

The first linear branch is defined by the Equations (6) and (9), while the post-cracking branch for $\sigma >\sigma_{cr}$, under the assumption of hyperbolic tension stiffening effect, can be defined with the following formulation up to the ultimate tensile strain of fabric $\epsilon_{f,u}$:

(12) $$\epsilon_{II,ts}=\frac{\sigma }{E_{f}}\left(1-\beta_{1}\beta_{2}{\left(\frac{\sigma_{cr}}{\sigma }\right)}^{2}\right)$$

where $\beta_{1}$ is assumed to be 0.5 in the case of no perfect bond at the fiber-matrix interface, in the post-cracking phase and $\beta_{2}$ equal to 1.0 in the case of short-term loads.

It should be noted that ACK and simplified models do not take into account directly the effect of matrix-to-fabric bond, as done by the tension stiffening model with the coefficient $\beta_{1}$. However, the ACK and simplified approach were calibrated on the basis of experimental results available in the literature at the time of the respective studies. As a consequence, the empirical expression Equation (3) implicitly takes into account the effect of bond for the average of experimental results.

## 3. Experimental Investigation

In this section, an experimental campaign for the mechanical characterization of a glass TRM composite is described. The experimental program was conducted at the Structure Laboratory of UNIPA, it provided tensile tests on composite strips of glass-TRM and mechanical characterization of their constituent materials. In particular, tensile tests are performed by using a clamp gripping method as recommended by the Italian guidelines [11]. In conclusion, the main results are presented and discussed.

### 3.1. Materials

**Figure 1:**
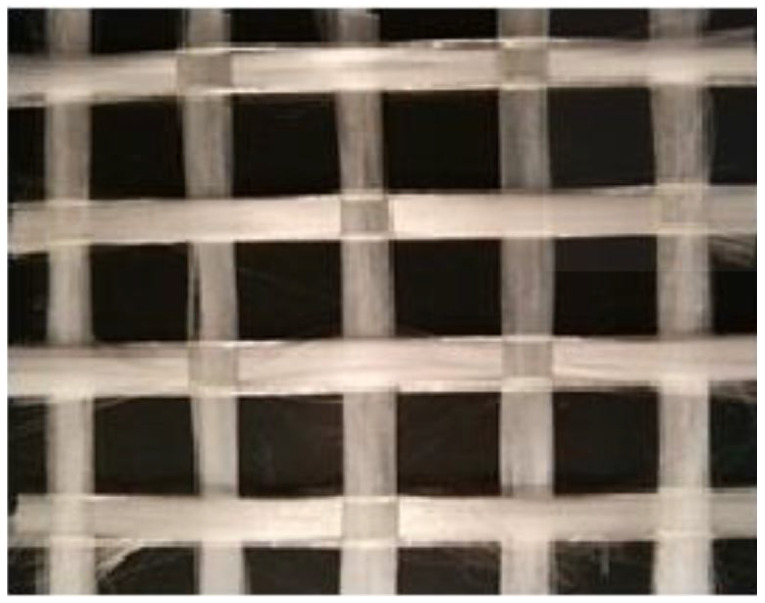
Alkali-resistant (AR) glass fiber with mesh size of 12 × 12 mm.

In this study, an alkali-resistant (AR) glass fiber mesh is adopted for the TRM system. The textile is manufactured in two orthogonal directions with a nominal spacing between bundles of 12 mm (Figure 1) and a nominal equivalent thickness of 0.088 mm. The unit weight and the density of the fibre are respectively 220 g/m^2^ and 2.5 g/cm^3^. The technical sheet of the producer provides a tensile strength of the glass fiber ≥ 1400 MPa and elastic modulus equal to 74 GPa.

The glass textile is coupled with a fibre-reinforced pozzolanic mortar based on hydraulic lime NHL5. Data sheet provided by the supplier declares a compressive strength ≥ 6.5 MPa and a flexural strength ≥ 3 MPa after 28 curing days.

**Figure 2:**
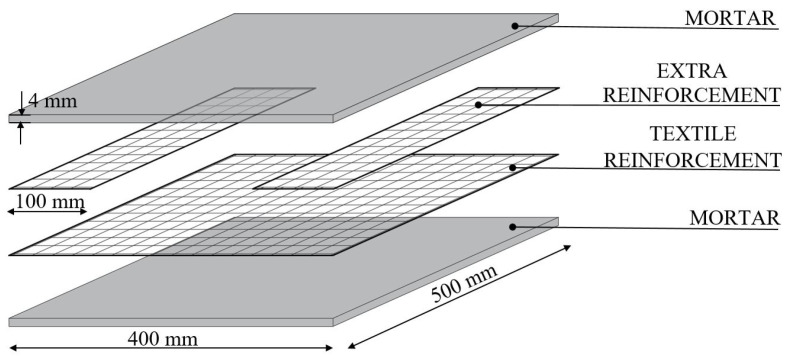
Geometry of glass-TRM (Textile Reinforced Mortar) panels.

A total of seven glass-TRM coupons (60 × 400 × 8 mm) are extracted from two panels with dimensions of 500 × 400 × 8 mm. TRM panels were manufactured following the American guidelines AC434.13 Annex A [12]. Firstly, a thin layer of mortar (about 4 mm) was cast, then glass fiber mesh was positioned and finally a second layer of mortar with same 4 mm thickness was applied. Moreover, an additional 100 mm width fibre band was applied at the ends of panel (Figure 2), in order to avoid premature ruptures of the samples in the gripping areas during the tests. A bi-component adhesion promoter was applied at the interfaces over the fabric in order to ensure a good adhesion between fiber and matrix in contact each other. Panels were cured in controlled environmental conditions, with a temperature of about 20 °C and 70% relative humidity for 28 days. After the curing period, the two panels were cut and two coupons were selected from the first one (TRM-1) and five from the second one (TRM-2).

### 3.2. Test Set-Up

Tests were addressed to investigate the tensile behaviour of the manufactured TRM composite.

Mechanical properties of glass textile are derived from tensile tests on four strips of fiber with dimension of 50 × 300 mm and five samples to scale with dimension of 40 × 230 mm, according to ISO 13934-1 standards [13]. Tensile tests of glass textile are performed in displacement control, with a rate equal to 20 mm/min. The force-elongation relationship is measured by the testing machine in all cases and an additional extensometer is added in 40 × 230 mm fiber specimens with a gauge length of 100 mm.

In order to verify the mechanical properties of the mortar matrix (NHL5), three-point bending tests and uniaxial compression tests are performed according to the standard UNI EN 1015-11 [14]. Six 40 × 40 × 160 mm prisms are cast and tested under flexure and subsequently compression, after 28 curing days.

**Figure 3:**
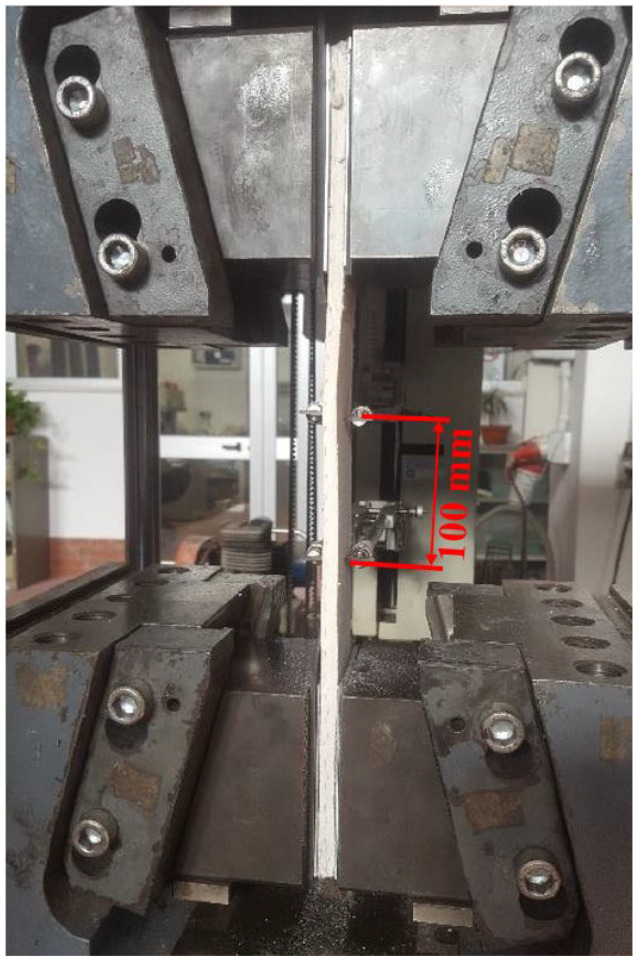
Set-up of uniaxial tensile test.

Glass-TRM systems are investigated by direct tensile tests on seven coupons (50 × 400 × 8 mm), two of these are cut from the first TRM panel (TRM-1) and five from the second one (TRM-2). The test set-up provides clamp gripping method by using an universal Zwick/Roell BTC-EXOPTIC.001 testing machine with force capacity equal to 600 kN. In particular, the tensile load is transferred from the test system through the clamp gripping wedges of the machine at the two ends of the specimen. The wedges applies a lateral pressure equal to 10 bar at the metal tabs, which are glued to the ends of each sample. According to this set-up, tests are carried out in displacement controlled mode with an elongation rate of 2.5 mm/min. Axial strains are recorded on a gauge length of 100 mm by means of two knife displacement transducers positioned in the middle part of specimen, as shown in Figure 3.

### 3.3. Results

Table 1 summarizes the main results obtained from fiber and matrix characterization tests. In particular, tensile tests on glass textile provide an average tensile strength $\sigma_{f}$ equal to 354 MPa and an elastic modulus $E_{f}$ of 28.4 GPa. It is stressed that the values resulting in the characterization tests are lower than that provided by the producer. Differently, tests on mortar samples provided an average flexural strength $\sigma_{mf}$ equal to 3 MPa and compressive strength $\sigma_{mc}$ equal to 10.6 MPa, in good agreement with the values in the data sheet.

**Figure 4:**
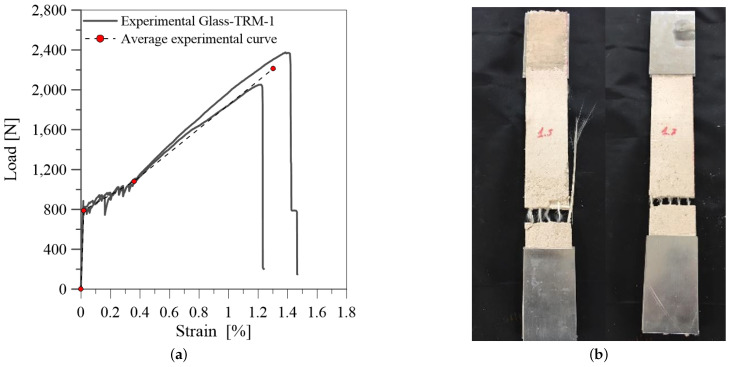
Tensile test on specimens TRM-1: (**a**) tensile load-axial strain curve; (**b**) failure mode.

**Figure 5:**
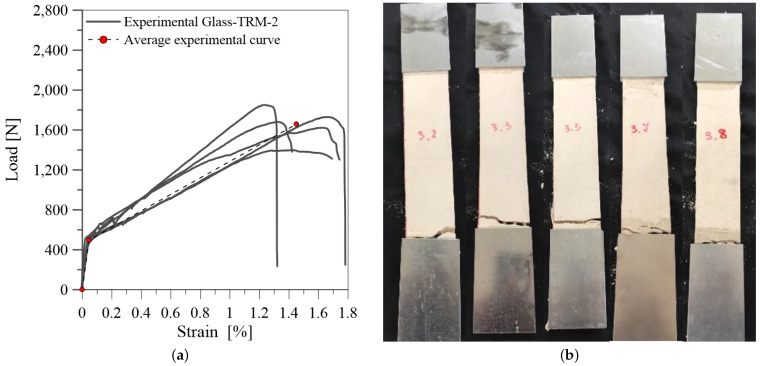
Tensile test on specimens TRM-2: (**a**) tensile load-axial strain curve; (**b**) failure mode.

Figure 4a and Figure 5a show the results of tensile tests on glass-TRM specimens in terms of tensile load-axial strain curves for the two series of specimens (TRM-1 and TRM-2) tested respectively after 71 and 64 curing days.

As shown in Figure 4a, specimens TRM-1 exhibit the characteristic trilinear trend, highlighted by the linearized average experimental curve (dashed line). The first linear ascending branch represents the uncracked stage; in this phase the composite behaviour depends mainly on the mortar properties. The second branch begins by reaching the first cracking load ($P_{1}$) and it is characterized by a significant decrease of the stiffness. It corresponds to the phase of crack propagation and depends on the quality of the bond between fiber and mortar. The third stage corresponds to the crack-stabilizing phase; the slope of this branch ($E_{3}$) depends mainly on mechanical properties of fiber, that in this stage is the only resisting phase.

Figure 5a shows the results for specimens TRM-2. In this case the trilinear trend is less evident. This can be attributed to a potentially compromised test due to some defects in the samples. After reaching the first crack load ($P_{1}$), few cracks evolved in the composite so the transient phase, between uncracked and cracked stage, is not visible. Consequently, the behaviour is immediately governed by the fabric properties and the fiber-matrix residual interaction. The clamping force induced a triaxial tension state close to the wedges; the stress concentration in the gripping area did not allow for the redistribution of the applied tension load and the fiber rupture occurred mostly within the clamped length, Figure 5b.

The experimental results of tensile tests on glass-TRM systems are summarized in Table 2. The load values P_1_, P_2_ and P_3_ are measured by the load cell of the machine while the corresponding axial strains $\epsilon_{1}$, $\epsilon_{2}$, $\epsilon_{3}$ are calculated through the measurements of the knife transducers along the central part of the strips. The slope values $E_{1}$, $E_{2}$, $E_{3}$ of the three branches are calculated by referring the tensile stress to the fiber cross section $A_{f}$, equal to 4.9 mm^2^, as indicated in the AC434.13 [12].

It should be observed that the results for the two groups of samples (TRM-1 and TRM-2) are quite different from each other.In particular, TRM-1 specimens achieve higher strength values than TRM-2 specimens. Moreover, different failure modes are identified. Matrix cracking occurs along the length of specimens coupled with slippage and fiber rupture for TRM-1 specimens (Figure 4b). Differently, failure at the clamping zones is observed in TRM-2 specimens, as evidenced in Figure 5b. The tensile behaviour of these specimens during the test was characterized by the slippage along the clamping zones, probably due to an inefficient gluing of the tabs to the specimens. Additionally, the fabric slippage proved to be more marked with resperct to the series TRM-1. This odd behaviour is probably due to the different manufacturing of the samples and to a slightly different composition of the mortar matrix. In fact, specimens of TRM-2 series were manufactured separately from TRM-1, leading to the observed experimental differences.

Considering the unexpected results discussed above, the data of specimens TRM-2 are not considered in the following for setting up the experimental database and providing an assessment of the existing analytical models.

## 4. Experimental Database

As discussed above, the typical behaviour of a TRM system under a tensile test can be idealized as a trilinear curve [15,16]. However, this kind of behaviour does not always occur, being dependant on the boundary conditions due to the adopted test set-up.

The experimental evidence shows the main difference by using clevis or clamping grip method to perform tensile tests on TRM strips. The clevis grip method, promoted by American Acceptance Criteria [12], provides to transfer the tensile load through metal plates glued on TRM coupons and connected to the machine with a pin. In this case, load is transferred by means of shear stress at the fiber-matrix interface and the failure occurs due to fiber slippage. As a consequence, the tensile strength of the fiber is never reached and specimens fail for lower load values [17] and the global response can be idealized as a bilinear curve. By contrast, the clamping grip method, originally proposed by Ascione et al. [18] and then included in the Italian Guidelines [11], allows a more complete mechanical characterization of the TRM composites (trilinear trend). In fact, in this case a lateral pressure applied to the gripping area, limits fiber-matrix slippage and it provides constituent materials to perform their mechanical performance. However, this conditions is not always reached. Despite the test set-up, an obvious slippage can arise due to the stress transfer at the interface, which features can be considered as a function of the nature of the fiber [19] and fiber treatment (i.e., coated or un-coated fiber) [20,21].

Results of experimental tensile tests conducted by several authors [9,19,20,21,22,23,24,25,26] are collected in Table 3, including the results of TRM-1 specimens of the current investigation. As discussed above, specimens of TRM-2 series are not considered due to the experimental problems highlighted by the results discussed in the previous section The database is set up by focusing on the failure modes of the TRM systems (A = slippage at clamps or cracking close to clamps; B = cracking along the specimen with tensile rupture of fiber; C = cracking along the specimen with slippage of fiber; D = cracking of matrix with subsequent fabric detachment) and on the influence of the main parameters such as the nature of the fiber, the mortar grade and the test set-up employed (CLAMP = clamping grip method, CLEV = clevis gripping method).

## 5. Comparison and Discussion

Experimental results in the literature are compared against the three available analytical models described in the previous sections. In the first part of this section, the experimental-theoretical comparison is shown in terms of load-strain curves, while in the second part a general quantitative evaluation is provided in terms of dissipated energy.

### 5.1. Tensile Load-Strain Curves

**Figure 6:**
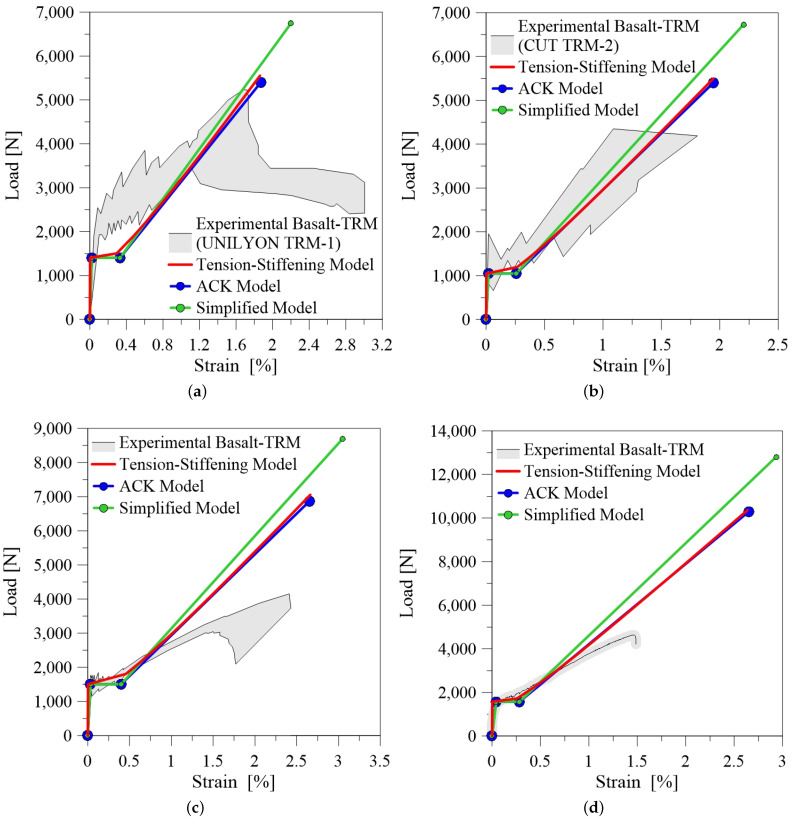
Comparison between experimental and analytical curves for basalt-TRM: (**a**) reference Lignola et al. [21] (Fabric Reinforced Cementitious Matrix (FRCM) 1 UNILYON); (**b**) reference Lignola et al. [21] (FRCM 2 CUT); (**c**) reference D’Anna et al. [24] (2L); (**d**) reference D’Anna et al. [24] (3L).

Figure 6 show some theoretical-experimental comparisons in terms of tensile load-axial strain curve for basalt-TRM. It is evident that the three models are generally capable of predicting the first peak load $P_{1}$ and the corresponding axial strain $\epsilon_{1}$, while in some cases Figure 6b,c) they are also suitable to predict the slope and the length of the second transient phase. Otherwise, the stiffness in the third branch is often overestimated, due to the fact that the analytical models are based on simplified hypotheses, which do not take into account the progressive rupture of filaments inside the rovings and the fiber-matrix residual interaction during the final phase of the test.

**Figure 7:**
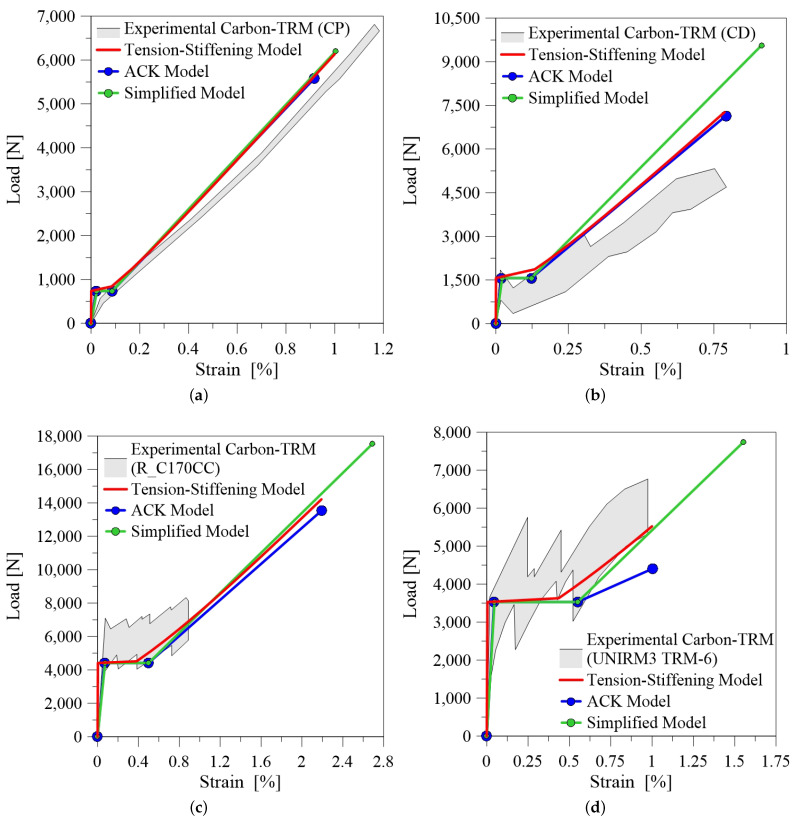
Comparison between experimental and analytical curves for carbon-TRM: (**a**) reference Bellini et al. [19] (CP); (**b**) reference Bellini et al. [19] (CD); (**c**) reference D’Antino and Papanicolaou [23] (R-C170CC); (**d**) reference Carozzi et al. [22] (FRCM 6 UNIRM3).

Figure 7 shows the theoretical-experimental comparisons for carbon-TRM. In this case, the analytical curves are in good agreement with the observed experimental trend. It is worth highlighting the influence of fibre treatment. Coated treatment of fiber bundles can reduce the thickness of the process area at the fiber-matrix interface. In the process area the matrix interacts with the outer layer of a single bundle of fiber, consequently when the load increases the inner core of the fiber slips causing the typical telescopic failure. As shown in Figure 7c, in some case this phenomena prevents to reach the mechanical performance of the fiber because the composite failures prematurely. It should also be made clear that the third branch is usually non-linear with evident loss of load when the core of each bundle detaches and slips from the outer layer.

**Figure 8:**
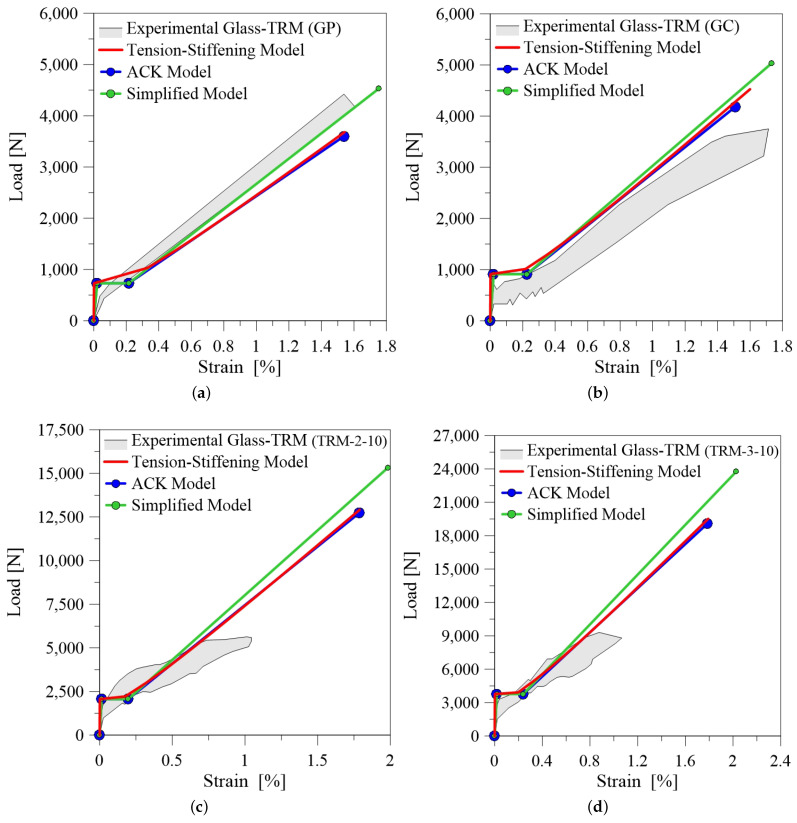
Comparison between experimental and analytical curves for glass-TRM: (**a**) reference Bellini et al. [19] (GP); (**b**) reference Bellini et al. [19] (GC); (**c**) reference Bramato et al. [20] (3L-5); (**d**) reference Bramato et al. [20] (3L-10).

The typical response of a tensile test recorded for glass-TRM is a bilinear load-axial strain curve, Figure 8. In this case the three models provide a second transition stage rather short. Despite they allow to grasp the slope of the third branch, the analytical prediction overestimates the ultimate elongation in the cases reported in Figure 8c,d.

**Figure 9:**
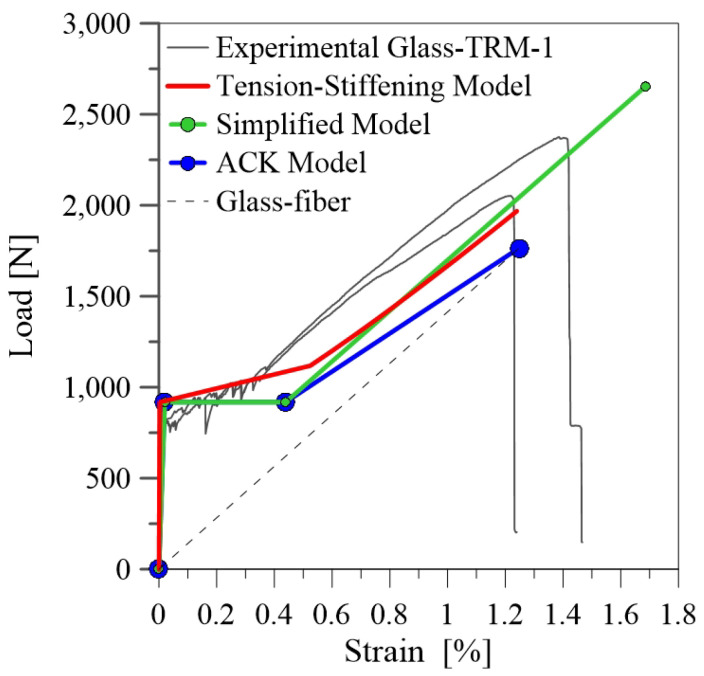
Comparison between current experimental results and analytical curves for TRM-1 specimens.

Figure 9 reports the comparison with the benchmark data of the current experimental study. Observing the results, it emerges that the first stage of the tensile load-axial strain curve is well reproduced and models provide overall a similar trend. The slope of the third branch is well predicted and almost parallel to the stiffness of the glass fiber fabric. Additionally, Tension Stiffening and ACK models predict with good accuracy the ultimate elongation. It is also worth noting that the reliability of the models is improved from the fact that the experimental results are in this case recorded from the internal transducer, providing a local measure of the axial strain. This fact highlights the importance of providing suitable measuring systems during the tensile testing of TRM composites. Further investigations should be addressed to provide an unified monitoring set-up for this kind of tests.

Generally, the three analytical models show a limited reliability in predicting the experimental results. The main disagreement is the overestimation of ultimate elongation and this is due probably to the fact that the measure of strains during a tensile tests in TRM composites is affected by the measuring system, which is variable in the tests presented in the literature. Moreover, these models work better with TRM composites that exhibit a trilinear tensile behaviour, i.e., specimens tested through direct clamping, while substantial differences can be observed when clevis system is adopted.

### 5.2. Statistical Evaluation

A general quantitative evaluation on the predictive efficiency of the models is made by comparing the analytical $E_{cr,analytical}$ and the experimental $E_{cr,exp}$ value of dissipated energy during the post-cracking stage. This last, $E_{cr,exp}$, is calculated as the area under the average experimental curve, and the results are summarized in Table 4.

**Figure 10:**
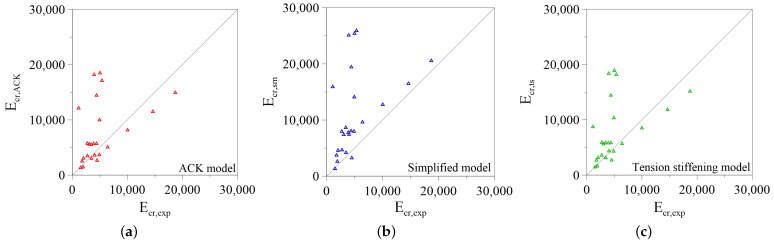
Comparison between analytical and experimental fracture energy: (**a**) Aveston–Cooper–Kelly (ACK) Model; (**b**) Simplified Model; (**c**) Tension Stiffening Model.

Two distinct comparisons are made in the current analysis: in the first, the dissipated energy is evaluated as the area under the overall effective curve, while in the second it is calculated by cutting the theoretical curve in correspondence of the ultimate value of axial strain deduced experimentally. This operation is made due to the evidence that models tend generally to overestimate the ultimate strain for several experimental results, as highlighted by Figure 10a–c. These last show the comparisons between the experimental and predicted dissipated energy in distinct graphical form for the three models analyzed.

It is evident that the models have similar predictive capability but the dispersion of the data seems to be quite wide. As discussed above, it is due to the fact that generally the models overestimate the ultimate elongation, and the measure of this last is strongly influenced by the testing apparatus and measuring system.

**Figure 11:**
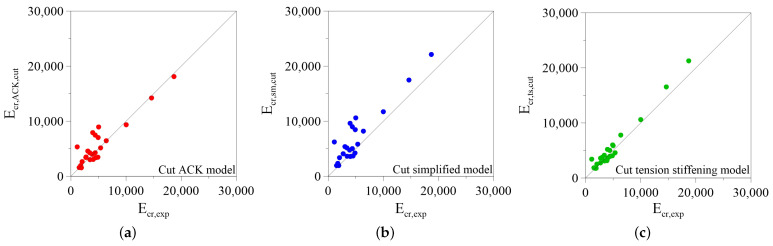
Comparison between experimental and analytical fracture energy, calculated by considering the maximum experimental elongation of the composite: (**a**) Cut ACK Model; (**b**) Cut Simplified Model; (**c**) Cut Tension Stiffening Model.

Similarly, Figure 11a–c show the validation of the models by considering the cut curves. In this case the ratios between experimental $E_{cr,exp}$ and the analytical $E_{cr,analytical,cut}$ values of the dissipated energy are closer to one, and the dispersion of the results reduces substantially with respect to the previous case.

**Figure 12:**
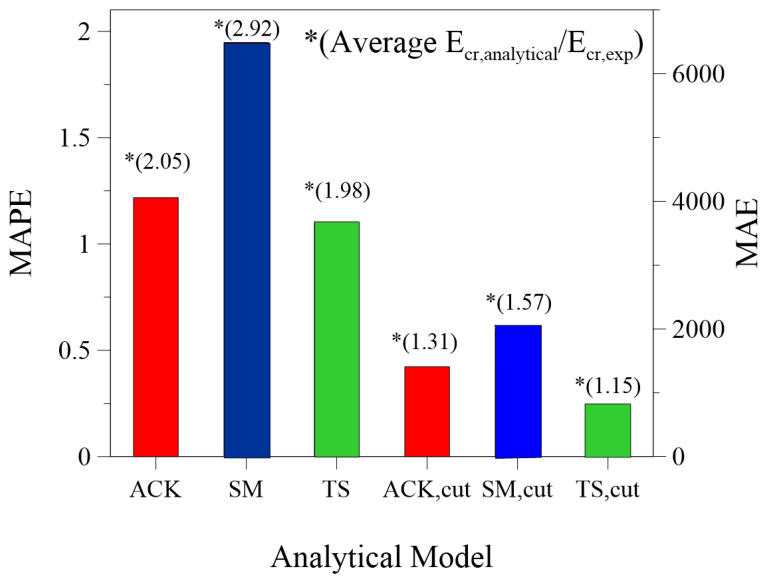
Mean Absolute Error (MAE) and Mean Absolute Percentage Error (MAPE) measures for the analytical models: ACK, SM, TS, ACK, cut, SM, cut, TS, cut.

Finally, Figure 12 resumes the performance of each analytical approach in predicting the dissipated energy in the post-cracking stage. It shows the value of the Mean Absolute Error (MAE) and the Mean Absolute Percentage Error (MAPE) for complete and cut curves, including the value of the average ratio $E_{cr,analytical}/E_{cr,exp}$. It is evident that the models without cutting substantially overestimate the fracture energy with great values of the error. If the observation is limited to the measured ultimate strain, the accuracy increases noticeably, and the average ratio drops down significantly. The tension stiffening model seems to work better with respect to other models, giving the lower values of errors and average predicting ratio closer to 1.

## 6. Conclusions

This paper presented a review on the state of research for TRM tensile behavior, with particular reference to the analytical models available in the literature to predict the mechanical response: ACK, Simplified and Tension Stiffening Model. An experimental campaign on glass-TRM was included in order to better understand the complex mechanisms involved in tensile characterization and enrich the available database of experimental results.

Data available in the literature show a wide dispersion of the results and a limited repeatability due to the numerous variables (test set-up, fiber nature, fiber treatment, mortar grade) to be taken into account for the mechanical characterization.

Generally, results indicated that:

- the typical trilinear trend was observed when clamping grip method was employed to perform tensile test;
- the bond strength at the fiber-matrix interface can influence the cracking phase after that first cracking load is achieved in the composite;
- for a low bond grade, fiber slippage within the matrix can occurs and consequently composite reaches rupture prematurely; in this case the tensile trend can be idealized as a bilinear curve;
- a low bond strength at the fiber-matrix interface can result from fiber nature or fiber treatment, indeed the latter affects the thickness of the process zone and the failure modes.

Overall, the three analytical models analyzed in this work allow to estimate with enough accuracy the tensile behavior of the TRM tests but with some limitations. Comparison between results allowed for drawing the following conclusions:

- among the three models, the Tension Stiffening Model gives the lower error;
- the energy calculated with the Simplified Model can be overestimated up to 14 times;
- the average difference with experimental results can be drastically reduced by considering the cut curves at the maximum experimental elongation of the composite: in this last case the models overestimate the energy of fracture $E_{cr}$ of 31%, 57% and 15% respectively for ACK, Simplified and Tension Stiffening model.

As a closing reminder, the current study investigated the behaviour under uniaxial tension, while further future investigations should be carried out to analyze the behaviour of TRM composites under bi/tri-axial states of stress. Obviously, in this last case the effect of cracking and the geometry of the fabric will play a more important role, which needs to be properly studied.

## Figures and Tables

**Table 1:** Average results for AR glass fiber and mortar NHL5.

Fiber			Mortar	
$\sigma_{f}$ **[MPa]**	$\epsilon_{f,u}$ **[%]**	$E_{f}$ **[GPa]**	$\sigma_{\mathrm{mf}}$ **[MPa]**	$\sigma_{\mathrm{mc}}$ **[MPa]**
354	1.3	28.4	3.0	10.6

**Table 2:** Results of tensile tests on glass-TRM systems.

Specimen	$P_{1}$ [N]	$\epsilon_{1}$ [%]	$E_{1}$ [GPa]	$P_{2}$ [N]	$\epsilon_{2}$ [%]	$E_{2}$ [GPa]	$P_{3}$ [N]	$\epsilon_{3}$ [%]	$E_{3}$ [GPa]
1_TRM-1	759.7	0.014	1093.4	1102.5	0.360	19.9	2375.9	1.386	24.9
2_TRM-1	824.3	0.024	693.9	1063.5	0.361	14.3	2052.4	1.220	23.1
Av.	792.0	0.019	1083.0	1102.5	0.360	17.1	2214.1	1.303	24.1
**CoV (%)**	(4.08)	(26.19)	(24.54)	(0.01)	(1.80)	(16.34)	(7.31)	(6.37)	(3.82)
1_TRM-2	420.1	0.034	257.3	-	-	-	1622.7	1.590	16.0
2_TRM-2	571.6	0.068	173.0	-	-	-	1682.9	1.326	18.3
3_TRM-2	530.1	0.041	264.7	-	-	-	1852.1	1.237	22.9
4_TRM-2	470.5	0.043	225.1	-	-	-	1398.5	1.429	13.8
5_TRM-2	496.5	0.040	259.2	-	-	-	1731.3	1.664	15.7
Av.	497.7	0.045	227.4	-	-	-	1657.5	1.4	17.1
**CoV (%)**	(10.35)	(26.42)	(15.56)	-	-	-	(9.04)	(10.99)	(18.21)

**Table 3:** Experimental database.

Ref.	ID Sample	Fiber		Mortar		Test Set-Up	Failure Mode
		Type	$E_{f}$ [GPa]	Type	$f_{\mathrm{mt}}$ [MPa]		
[9]	TB1	Basalt	67	cement-based	2.48	CLAMP	B
	TB2	Basalt	67	cement-based	2.48	CLAMP	B
	TB3	Basalt	67	cement-based	2.48	CLAMP	B
	TB4	Basalt	67	cement-based	2.48	CLAMP	B
[21]	FRCM 1	Coated basalt	110	cement-based	0.80	CLEV	A/B
	FRCM 1	Coated basalt	116	cement-based	1.50	CLAMP	C
	FRCM 1	Coated basalt	111.5	cement-based	1.30	CLAMP	B
	FRCM 2	Coated basalt	111.5	lime-based	1.20	CLAMP	C/B
[24,26]	1L	Basalt	83	cement-based	2.00	CLAMP	B
	2L	Basalt	83	cement-based	2.00	CLAMP	B
	3L	Basalt	83	cement-based	2.00	CLAMP	B
[19]	CP	Carbon ${}^{\left(*\right)}$	240	hydraulic lime-based	1.92	CLAMP	A/B
	CD	Carbon	240	hydraulic lime-based	2.91	CLAMP	A/B
	GP	Glass ${}^{\left(*\right)}$	65	hydraulic lime-based	1.92	CLAMP	A
	GC	Coated glass	70	hydraulic lime-based	2.00	CLAMP	A/B
[23]	R-C170CC	Coated carbon	219	cement-based	3.35	CLAMP	A/C
	2L-10	Coated glass	108	lime-based	1.10	CLAMP	B
	3L-10	Coated glass	108	lime-based	1.50	CLAMP	B
[22]	FRCM 6	Coated carbon	187	cement-based	3.35	CLAMP	A/B
	FRCM 6	Coated carbon	219	cement-based	4.00	CLAMP	C
[25]	FT	Coated glass	66.21	cement-based	0.80	CLEV	C
current exp.	TRM-1	Glass ${}^{\left(*\right)}$	28.4	hydraulic lime-based	2.00	CLAMP	C/B

${}^{\left(*\right)}$ Adhesion promoter.

**Table 4:** Experimental-theoretical comparison.

Ref.	ID Sample	Effective Curve			Cut Curve		
		$\frac{E_{cr,ACK}}{E_{cr,exp}}$	$\frac{E_{cr,sm}}{E_{cr,exp}}$	$\frac{E_{cr,ts}}{E_{cr,exp}}$	$\frac{E_{cr,ACK,cut}}{E_{cr,exp}}$	$\frac{E_{cr,sm,cut}}{E_{cr,exp}}$	$\frac{E_{cr,ts,cut}}{E_{cr,exp}}$
[9]	TB1	0.79	1.50	0.89	1.01	1.28	1.22
	TB2	0.81	1.27	0.85	0.93	1.17	1.06
	TB3	0.79	1.13	0.81	0.97	1.19	1.13
	TB4	0.80	1.10	0.81	0.97	1.18	1.14
[21]	FRCM 1	1.84	2.42	1.83	1.49	1.77	1.24
	FRCM 1	1.29	1.84	1.33	0.98	1.15	0.89
	FRCM 1	2.12	2.94	2.16	1.27	1.51	1.02
	FRCM 2	1.47	2.01	1.49	1.03	1.23	0.81
[24,26]	1L	1.61	2.53	1.70	1.25	1.51	1.21
	2L	2.02	2.85	2.09	1.43	1.72	1.22
	3L	3.28	4.42	3.29	1.70	2.04	1.14
[19]	CP	0.60	0.72	0.60	0.75	0.82	0.88
	CD	1.50	2.22	1.53	1.31	1.65	1.23
	GP	0.87	1.21	0.90	0.87	1.05	0.90
	GC	1.26	1.70	1.31	1.29	1.49	1.31
[23]	R-C170CC	3.19	4.83	3.39	0.96	1.09	0.85
[20]	3L-5	4.59	6.33	4.63	2.00	2.42	1.31
	2L-10	10.81	14.17	7.75	4.77	5.55	3.05
	3L-10	3.70	5.08	3.78	1.79	2.12	1.17
[22]	FRCM 6	0.89	1.85	1.08	0.76	0.89	0.90
	FRCM 6	0.76	1.63	0.88	0.71	0.86	0.83
[25]	FT	1.43	2.12	1.51	1.09	1.33	1.01
current exp.	TRM-1	0.76	1.36	0.85	0.80	1.00	0.91

## Data Availability

The data that support the findings of this study are available from the corresponding author upon reasonable request.
